# A Case for Thalamic Mechanisms of Schizophrenia: Perspective From Modeling 22q11.2 Deletion Syndrome

**DOI:** 10.3389/fncir.2021.769969

**Published:** 2021-12-08

**Authors:** Yanbo Jiang, Mary H. Patton, Stanislav S. Zakharenko

**Affiliations:** Division of Neural Circuits and Behavior, Department of Developmental Neurobiology, St. Jude Children’s Research Hospital, Memphis, TN, United States

**Keywords:** schizophrenia, thalamocortical, thalamus, 22q11 deletion syndrome, hallucinations

## Abstract

Schizophrenia is a severe, chronic psychiatric disorder that devastates the lives of millions of people worldwide. The disease is characterized by a constellation of symptoms, ranging from cognitive deficits, to social withdrawal, to hallucinations. Despite decades of research, our understanding of the neurobiology of the disease, specifically the neural circuits underlying schizophrenia symptoms, is still in the early stages. Consequently, the development of therapies continues to be stagnant, and overall prognosis is poor. The main obstacle to improving the treatment of schizophrenia is its multicausal, polygenic etiology, which is difficult to model. Clinical observations and the emergence of preclinical models of rare but well-defined genomic lesions that confer substantial risk of schizophrenia (e.g., 22q11.2 microdeletion) have highlighted the role of the thalamus in the disease. Here we review the literature on the molecular, cellular, and circuitry findings in schizophrenia and discuss the leading theories in the field, which point to abnormalities within the thalamus as potential pathogenic mechanisms of schizophrenia. We posit that synaptic dysfunction and oscillatory abnormalities in neural circuits involving projections from and within the thalamus, with a focus on the thalamocortical circuits, may underlie the psychotic (and possibly other) symptoms of schizophrenia.

## Introduction

Schizophrenia is a devastating neurodevelopmental disorder affecting 0.7% of the world’s population (Tandon et al., 2008). The disorder is characterized by a range of symptoms categorized into three primary groups: positive, negative, and cognitive (Liddle, 1987). Symptoms arise during late adolescence or early adulthood, between the ages of 16 and 30 years, and they typically appear earlier in men than in women (Mueser and McGurk, 2004; APA, 2013; Charlson et al., 2018). Positive symptoms, which include delusions and hallucinations, can often be ameliorated by antipsychotics. However, those medications are associated with severe side effects, and they have limited efficacy and ability to significantly improve cognitive or negative symptoms. Therefore, symptoms persist with varying degrees of severity throughout the patient’s lifetime and lead to an approximately 15-year shorter life expectancy and higher risk of suicide (Mueser and McGurk, 2004; Tanskanen et al., 2018). Despite decades of research into the etiology and pathogenesis of schizophrenia, the mechanistic understanding of the disease remains in its infancy. Both genetic and environmental factors appear to play a role in the etiology of schizophrenia (reviewed in Lewis and Lieberman, 2000; Mueser and McGurk, 2004). It has become clear that schizophrenia is a highly heritable, polygenic disease that affects multiple neural circuits throughout the brain. The complex etiology and symptom diversity of schizophrenia make it difficult to model, resulting in a dearth of valid animal models.

In this review, we propose that many of the diverse symptoms of schizophrenia are attributable to pathology of a single brain region, the thalamus. Our hypothesis is supported by multiple clinical observations of thalamic abnormalities in individuals who had schizophrenia and recent findings of thalamocortical disruption in mouse models carrying a genetic lesion strongly associated with schizophrenia in humans. We also discuss the anatomical and functional connections between various thalamic nuclei and other brain regions and describe how these connections enable the thalamus to broadcast information from a single modality to a wide range of cortical and subcortical regions via recurrent connections. We then identify the “hot spots” within these structures and pathways that are associated with schizophrenia and propose the hypothesis that disruptions in thalamic circuits may underlie a range of schizophrenia symptoms.

## Diversity of Schizophrenia Symptoms and Treatments

The positive symptoms of schizophrenia, which are frequently referred to as the psychotic symptoms, include delusions and hallucinations. Delusions are strong rigid beliefs that persist despite the existence of conflicting evidence, and hallucinations are vivid, perception-like experiences that occur without incoming sensory stimuli. Hallucinations occur in multiple sensory modalities, and auditory hallucinations (frequently auditory verbal hallucinations) are the most common (Mueser and McGurk, 2004). Positive symptoms are present in 60–90% of cases (Bauer et al., 2011), are self-reported, may last from minutes to hours, and in the majority (∼70%) of cases are alleviated by antipsychotics (Goghari et al., 2013; Bohlken et al., 2017) that inhibit D2 dopamine receptors (DRD2s) (Seeman and Lee, 1975; Carlsson, 1988). Negative symptoms denote the deficit or lack of normal behaviors and fall into two major categories: reduction in the expression of emotions or speech and avolition or apathy. Within the second category, amotivation, anhedonia, and asociality may be demonstrated. Negative symptoms are correlated with poor functional outcome in individuals with schizophrenia and are less likely to be alleviated by drug therapy (Fervaha et al., 2015; Fusar-Poli et al., 2015). Lastly, cognitive symptoms are defined by and include abnormal thinking, poor problem-solving skills and decision-making abilities, the inability to communicate effectively, and deficits in attention and working memory. For a more nuanced description of schizophrenia symptoms see Lewis and Lieberman (2000) and Mueser and McGurk (2004).

The most common and efficacious treatment options for schizophrenia are pharmacological interventions (Miyamoto et al., 2012). Antipsychotics (also known as neuroleptic agents) comprise a broad category of medications initially developed to treat psychosis. First-generation antipsychotics (e.g., haloperidol) were introduced in the mid-20th century, act primarily at DRD2s, and mostly alleviate positive symptoms (Seeman, 1992; Leucht et al., 2003; Miyamoto et al., 2005). This treatment advance led to the dopamine hyperactivity hypothesis of schizophrenia, which was formulated in the 1960s, after the discovery of the antipsychotic actions of chlorpromazine (reviewed in Baumeister and Francis, 2002; Carlsson et al., 2004). First-generation antipsychotics can cause debilitating extrapyramidal motor side effects (e.g., tardive dyskinesia, rigidity, dystonia, and tremor), hyperprolactinemia, neuroleptic malignant syndrome, and prolongation of the QT interval, among others. Consequently, more effective treatment options were needed. In response, second-generation (or atypical) antipsychotics were developed to alleviate symptoms more effectively with fewer and less severe side effects.

Second-generation antipsychotics demonstrate DRD2 antagonism and target serotonin (5-HT_2__*A*_), norepinephrine (α, β), and glutamatergic (NMDA) receptors (Meltzer et al., 1989; Horacek et al., 2006). These drugs are often a more effective option for individuals with schizophrenia who do not respond to first-generation antipsychotics or who experience significant extrapyramidal motor side effects. For example, clozapine is uniquely effective in treatment-resistant schizophrenia. However, the effects of second-generation antipsychotics on cognitive symptoms and negative symptoms are still less than ideal, and their use is mired with side effects such as weight gain and its metabolic effects, hypotension, sedation, and anticholinergic symptoms (Üçok and Gaebel, 2008; Leucht et al., 2013).

As many as one-third of individuals with schizophrenia do not respond to any pharmacologic treatments (Brenner et al., 1990). In those cases, non-pharmacologic treatment options may more effectively reduce positive symptoms and negative symptoms, though they are imprecise and non-specific to affected neural circuits. Non-pharmacologic approaches include neuromodulation therapies, such as transcranial magnetic stimulation, electroconvulsive therapy, and deep-brain stimulation (Dokucu, 2015). Finally, cognitive training can improve auditory gating and working memory deficits (Genevsky et al., 2010; Popov et al., 2011; Haut et al., 2017).

Novel treatment options for schizophrenia are continuously being developed. Strategies include adjusting agonism or antagonism at various receptors to increase drug efficacy and decrease side effects. Various brain-stimulation approaches are being explored, and while such approaches have some merit, the field desperately needs more advanced, mechanism-based strategies.

The divergent effects of antipsychotics on the wide range of schizophrenia symptoms and the multitude of the disorder’s seemingly unrelated symptoms support the possibility that multiple genetic and/or environmental insults cause distributed network deficits that culminate in the disease. Alternatively, the diverse symptoms could arise from defects in a common neural hub that sends projections to multiple brain regions that are each responsible for distinct behaviors. This last consideration calls for a model that posits a common pathogenic disruption in a central hub that serves as the molecular or cellular mechanism underlying the majority (or at least a substantial subset) of schizophrenia symptoms. The thalamus may be that vulnerable central hub, and we suggest that thalamic damage alone can explain the diversity of schizophrenia symptoms.

## Thalamic Abnormalities in Schizophrenia

The thalamus can be divided into separate nuclei that have distinct patterns of connections with the cortex, subcortical brain structures, and sensory systems. Here we discuss aspects of thalamic function in the context of schizophrenia; a more comprehensive description of the anatomy and function of the thalamus is beyond the scope of this work and is reviewed elsewhere (Jones, 2007; Sherman and Guillery, 2013). Converging evidence indicates that thalamic abnormalities are central to schizophrenia pathophysiology, from the early course of the disorder to the chronic stages, contributing to clinical symptoms and cognitive and sensory deficits (reviewed in Cronenwett and Csernansky, 2010; Steullet, 2020). Indeed, thalamic disruptions in individuals with schizophrenia are well documented (Byne et al., 2009; Adriano et al., 2010; Spoletini et al., 2011; Haijma et al., 2013); these disruptions range from the cellular level to the structural and functional levels. In postmortem examination of the brains of people who had schizophrenia, various thalamic nuclei show decreased cell counts (Pakkenberg, 1990, 1992; Young et al., 2000; Byne et al., 2002; Danos et al., 2005). Blood flow is decreased in thalamic nuclei during behavioral tasks (Heckers et al., 2000), and the overall thalamic volume is decreased in individuals with schizophrenia (Danos et al., 2003; Steullet, 2020). Furthermore, the rate and frequency of hallucinations are correlated with thalamic volume reduction (Neckelmann et al., 2006; Huang et al., 2015). Collectively, these data suggest that a disruption of information flow to and from the thalamus gives rise to some symptoms of schizophrenia. In support of this notion, schizophrenia-like symptoms emerge when lesions selectively damage the thalamus while sparing the rest of the brain. For instance, strokes restricted to the thalamus are associated with sensorimotor and cognitive deficits similar to those observed in individuals with schizophrenia, including executive dysfunction, intrusion of unrelated ideas, poverty of speech, and anterograde amnesia (Schmahmann, 2003; Carrera and Bogousslavsky, 2006). Hallucinations and impaired reality monitoring have been seen in individuals with peduncular hallucinosis, which is caused by acute lesions restricted to the thalamus, midbrain, or pons (Benke, 2006). Individuals with peduncular hallucinosis have no prior history of psychiatric disease, but almost immediately after the lesion occurs, they experience complex scenic and stereotyped visual and auditory hallucinations that may last intermittently for several minutes (Benke, 2006). Furthermore, one report indicates that individuals with schizophrenia have abnormally high levels of dopamine in the thalamus (Oke et al., 1988), which is consistent with the dopamine hyperactivity hypothesis (Carlsson, 1988). However, because the thalamus normally contains very low levels of dopamine or its receptors, thalamic dopaminergic abnormalities in schizophrenia are rarely investigated and more evidence is needed to support this finding.

Abnormal connectivity of the thalamus is another feature of schizophrenia, as atypical morphology in the thalamus can affect downstream targets. For instance, a reduction in volume in cortical regions targeted by thalamic projections (thalamocortical projections) is observed in individuals with schizophrenia (Lewis et al., 2001). Currently, structural and functional disconnectivity of thalamocortical projections is considered a reliable phenotype of schizophrenia (Welsh et al., 2010; Marenco et al., 2012; Woodward et al., 2012). There are reports of reduced structural connectivity between the thalamus and cortical prefrontal areas and increased connectivity with somatomotor, somatosensory, and occipital cortices (Marenco et al., 2012; Woodward et al., 2012; Giraldo-Chica et al., 2018; Zhang et al., 2021), as well as a reduction in functional connectivity of the thalamus with the prefrontal and cingulate cortices (Tu et al., 2013; Anticevic et al., 2014; Giraldo-Chica and Woodward, 2017; Avram et al., 2018; Penner et al., 2018; Skåtun et al., 2018) and an increase in functional coupling between the thalamus and sensorimotor cortices have also been reported in individuals with chronic schizophrenia (Skudlarski et al., 2010; Anticevic et al., 2014; Giraldo-Chica and Woodward, 2017; Skåtun et al., 2018). Similarly, reduced functional connectivity between the thalamus and other targets, such as the striatum, are present in different stages of psychosis (Woodward and Heckers, 2016; Martino et al., 2018). Because the evidence for abnormal thalamocortical connections is so overwhelming (Silbersweig et al., 1995; Ferrarelli and Tononi, 2011; Smith et al., 2011; Sodhi et al., 2011), some researchers have suggested that schizophrenia is a disease of disrupted thalamocortical neuronal networks (Cronenwett and Csernansky, 2010). Noteworthy, a local ischemic infarction that disrupted neural connections emanating from the auditory part of the thalamus and projecting to the auditory cortex (auditory thalamocortical projections) in a previously non-psychotic individual with no prior neurological symptoms caused transient auditory (musical) hallucinations that occurred acutely postinfarction (Woo et al., 2014). The hallucinations were intermittent, lasted several minutes, occurred when the environment was quiet, and discontinued or reduced in volume when the individual was engaged in conversation. Together, these works point to the thalamus, especially thalamocortical projections, as the brain structure that is particularly vulnerable in schizophrenia.

Thalamic nuclei are functionally classified based on the origin of their synaptic inputs. For instance, nuclei that receive inputs from peripheral sensory areas are termed “first-order thalamic nuclei” and relay sensory signals to layer 4 cortical neurons within the respective cortical regions. Recurrent cortical circuits, or loops, contain local excitatory connections that most likely amplify thalamic inputs (reviewed in Sherman, 2001; Jones, 2002; Sherman and Guillery, 2002). After processing within the cortical circuits, pyramidal neurons in deep cortical layers (e.g., layer 6) project back to excitatory thalamic relay neurons and inhibitory neurons in the thalamic reticular nucleus (TRN), a shell-like structure that sits on the lateral edge of the thalamic nuclei (Hackett, 2007). The TRN is composed of a dense layer of GABAergic neurons and provides feed-forward inhibition to first-order excitatory relay neurons, thereby dampening their output (reviewed in Jones, 2002). However, within the cortico-thalamo-cortical loop, layer 5 cortical neurons also relay primary sensory information arising from the thalamus to second- or higher-order thalamic nuclei. This anatomical organization creates an interconnected hierarchical network of thalamocortical circuits or loops (Shepherd and Yamawaki, 2021) that are involved in various oscillatory activities in the brain. The more nuanced views and current perspectives on connectivity, function, and diversity of thalamocortical circuits and first-order and higher-order thalamic nuclei are described and reviewed elsewhere (Clascá et al., 2012; Kuramoto et al., 2015; Rikhye et al., 2018b; Halassa and Sherman, 2019; Mukherjee et al., 2020).

There is considerable evidence of abnormal oscillatory properties of the brain in schizophrenia. For instance, a decrease in gamma-frequency oscillations (30–80 Hz) and an increase in low-frequency oscillations, including delta oscillations (1–4 Hz) and theta oscillations (5–10 Hz), have been reported (reviewed in Lisman, 2012). However, only the abnormal increase in low-frequency oscillations (which normally occur during slow-wave sleep and are thought to arise through an interaction between the thalamus and the cortex; Llinas et al., 1999) are seen during the awake state in individuals with schizophrenia but not in their unaffected family members (Galderisi et al., 2009; Siekmeier and Stufflebeam, 2010; Lehmann et al., 2014). Significant correlations have been found between the increase in delta oscillations and both positive and negative symptoms of schizophrenia (Fehr et al., 2001, 2003). Furthermore, animal studies show that DRD2 antagonists block abnormal delta oscillations (via depolarization of thalamic cells), perhaps explaining the therapeutic action of antipsychotics (Zang, 2009). Together, these findings led to the hypothesis that elevated delta oscillations are a potential marker for the disease and specifically for hallucinations (Lisman, 2016).

Multiple attempts have been made to build a theoretical framework for the occurrence of hallucinations. Some theories have argued that the brain generates an internal model of the world to fit incoming sensory information; what is being experienced is the result of this interaction rather than sensory information alone (Picton and Stuss, 1994; Behrendt, 2003). Llinas and Pare (1991) suggest that conscious perception is encoded by intrinsic oscillatory activity in thalamocortical circuits. They postulate that most connectivity in thalamocortical circuits is geared toward generating internal activity that operates in the presence or absence of sensory input. The general prediction of this theory is that thalamocortical circuits are intrinsically active, and sensory input only modulates this activity (reviewed in Behrendt, 2003; Behrendt and Young, 2004). According to this theory, normal perception, dream imagery, and hallucinations differ only with respect to the degree to which they are constrained by external sensory stimulation. Hallucinations may be conceptualized as perception in the state of wakefulness that is under-constrained by sensory input (Llinas and Pare, 1991; Behrendt, 2003; Behrendt and Young, 2004). The prediction of these theories is that hallucinations are caused by impairments in thalamocortical afferents to sensory cortices (Gruzelier, 1999). Due to the strong interconnectivity of the thalamus to various cortical and subcortical regions, it is tempting to assume that other symptoms of schizophrenia may occur in a similar fashion, i.e., by abnormal oscillations in thalamic projections to other brain regions.

## Hypothesis: The Thalamus as the Central Node for Schizophrenia Symptoms

Thalamic nuclei canonically serve as relay stations that convey signals between brain regions and support oscillatory activity. Based on anatomic and histologic criteria, the thalamus can be grossly divided into four regions: the anterior group, medial division, lateral division, and posterior group. Each thalamic region can be further divided into multiple specific nuclei (reviewed in Rikhye et al., 2018b; Halassa and Sherman, 2019), and each thalamic nucleus, except the TRN, sends excitatory projections to downstream targets. The TRN provides local inhibition and serves as “the gatekeeper” to orchestrate the flow of signals from thalamocortical, thalamostriatal, and other thalamic projections (Jones, 2002; Steullet, 2020). Unsurprisingly, TRN dysfunction has been implicated in the neurobiology of schizophrenia (Ferrarelli and Tononi, 2011).

**FIGURE 1:**
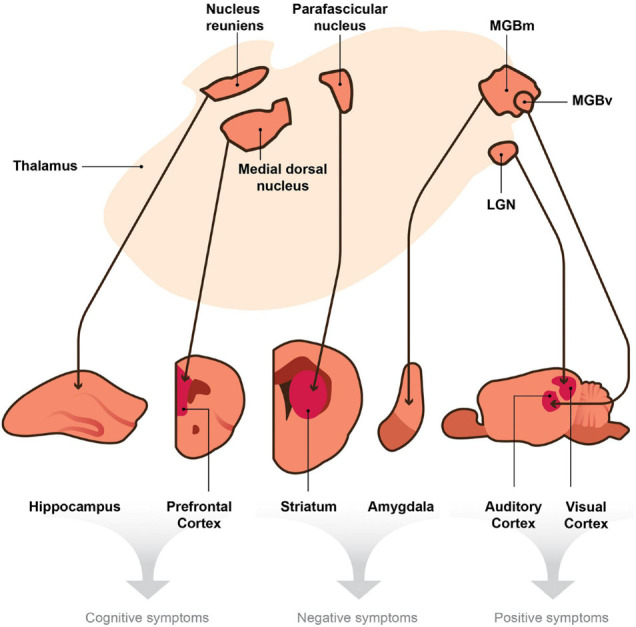
A model depicting the hypothesis that the thalamus is a central node within the brain that sends divergent projections to multiple targets involved in the generation of distinct behaviors. Thalamic nuclei project to cortical and subcortical efferent brain regions. We posit that thalamic disruptions, which in turn lead to deficits in signaling between the thalamo-effector pathways (thin arrows), cause the seemingly unrelated symptoms of schizophrenia. According to this hypothesis, disruptions in signaling or oscillatory activity between the nucleus reuniens and hippocampus and those between the medial dorsal nucleus and the prefrontal cortex could generate cognitive symptoms of schizophrenia (e.g., deficits in attention and working memory). Disruptions within the parafascicular nucleus or in its projections to the striatum and those in the medial division of the medial geniculate body (MGBm) and its projections to the amygdala may cause negative symptoms (e.g., avolition or blunted affect). Finally, aberrant signaling from the ventral region of the medial geniculate body (MGBv) to the auditory cortex and that from the lateral geniculate nucleus (LGN) to the visual cortex may produce positive symptoms of schizophrenia (e.g., auditory or visual hallucinations). Although the collateral projections of the TRN may play a role in these mechanisms, they are not included in this diagram for simplicity.

Thalamic nuclei project to different target regions in the brain (e.g., sensory cortices, prefrontal cortex, striatum, hippocampus, amygdala, etc.), and each target is responsible for different behaviors, such as arousal, language, sensory perception, motor function, executive functions, mood and motivation, etc. (Vertes et al., 2015). If thalamic projections connected to these targets are damaged, then different schizophrenia symptoms could arise (e.g., auditory and visual hallucinations, amotivation, cognitive deficits, etc.). We, therefore, posit that the thalamus serves as a central hub, and if damaged, it may play a unique and essential role in generating dissimilar, seemingly unrelated symptoms of schizophrenia (Figure 1).

## Hypothesis Testing in Animal Models of Schizophrenia

Gaining not only a thorough appreciation for the role of thalamic dysfunction in schizophrenia but also a more complete understanding of the disease as a whole requires the use of relevant animal models. Our current lack of mechanistic understanding of schizophrenia is partially due to the dearth of comprehensive models of the disease. As a result, we have no clear unified theory of the disease etiology, pathogenesis, reliable endophenotype, or biomarkers.

The advantages and disadvantages of using animal models in psychiatric research continue to be debated (Nestler and Hyman, 2010; Kaiser and Feng, 2015; Canetta and Kellendonk, 2018). Nonetheless, rodent studies have become instrumental for identifying neuronal circuit mechanisms underlying behaviors that are affected in psychiatric disorders. Existing animal models of schizophrenia are categorized into four primary classes (see Jaaro-Peled et al., 2010 for review). (1) *Neurodevelopmental models.* In these models, animals are exposed to various adverse events during gestation, the early postnatal period, or adolescence. The adverse events include maternal stress, maternal immune activation, maternal exposure to MAM (mitotoxin methylazoxymethanol acetate), and postnatal social isolation, including early postnatal maternal deprivation (Stilo and Murray, 2019). Neurodevelopmental models are based on the notion that environmental factors, though not necessarily causative, can influence the likelihood of disease by mimicking disruptions in brain development during critical periods, such as early life and adolescence. (2) *Pharmacological models*. These models mimic known disruptions within various neurotransmitter systems, specifically dopaminergic- and glutamatergic-signaling pathways. Chronic amphetamine administration blocks dopamine reuptake, thereby causing hyperactivity of the dopamine system (Seiden et al., 1993). Acute PCP or ketamine administration, which are both antagonists of the glutamatergic NMDA receptor, can induce positive symptoms (Krystal et al., 1994; Enomoto et al., 2007), though the symptoms do not accurately phenocopy schizophrenia symptoms. For instance, visual disturbances induced by systemically administered ketamine are relatively rare in schizophrenia, and auditory verbal hallucinations, which are more common in chronic schizophrenia, are rarely induced by ketamine (reviewed in Frohlich and Horn, 2014). (3) *Lesion models.* The most common lesion model in rodents is the neonatal ventral hippocampal lesion, which corresponds to the anterior hippocampus of primates (Lipska et al., 2003). This type of model results in reduced expression of the dopamine transporter, aberrant cortical pyramidal cell responses to dopamine signaling (Tseng et al., 2007), and a number of phenotypes resembling positive, negative, and cognitive symptoms (see Lipska, 2000 for review). (4) *Genetic models*. Heritability, or the proportion of the variability of a trait that is attributed to genetic inheritance, is as high as 80% in schizophrenia (Owen et al., 2016; see Legge et al., 2021 for review). Thus, genetic models of the disorder are important tools for gaining a better understanding of the underlying genetic causes of the symptoms of schizophrenia. Our understanding of the genetic underpinning of schizophrenia has greatly improved over the past decade. Several collaborative efforts have identified hundreds of common risk variants, rare variants (reviewed in Hyman, 2021; Legge et al., 2021), and copy number variants (CNVs) associated with schizophrenia (reviewed in Cook and Scherer, 2008). In 2014, one of the largest genome-wide association studies (GWAS) identified more than 100 common genetic variants (or single-nucleotide polymorphisms) in 108 loci associated with schizophrenia (Ripke et al., 2014). A Subsequent study has increased this number to 270 distinct loci in 130 genes (Legge et al., 2021). Unlike common risk variants, schizophrenia-associated CNVs are rare and generally confer a substantially higher risk of disease. In aggregate, common risk variants carry the greatest genetic risk for schizophrenia or other psychiatric disorders; however, individually, they have far less penetrance than do CNVs (by some estimates < 100 fold), making it highly improbable that a single common risk variant could produce robust, disease-relevant phenotypes in a transgenic animal (Hyman, 2021).

Copy number variants that are either deletions (del) or duplications (dup) of sections of the genome, ranging from dozens of base pairs to megabases spanning multiple genes, have been consistently implicated in the etiology of schizophrenia (International Schizophrenia Consortium, 2008; Stefansson et al., 2008; Xu et al., 2008; Malhotra and Sebat, 2012). For instance, the first large microdeletion identified as a risk for schizophrenia was found on chromosome 22q11.2 (reviewed in McDonald-McGinn et al., 2015). Schizophrenia develops in approximately 25% of carriers of this microdeletion (Karayiorgou et al., 2010; Van et al., 2017), and the risk of psychosis increases up to 41% in adults older than 25 years (Schneider et al., 2014), making this CNV one of the strongest genetic predictors of schizophrenia. In comparison, monozygotic twins of individuals with schizophrenia have a 40–50% risk of the disease (Ragland et al., 1992). Unlike common genetic variants, CNVs are relatively rare. For instance, 22q11.2 microdeletions account for 1–2% of schizophrenia cases (Bassett and Chow, 2008; International Schizophrenia Consortium, 2008; Stefansson et al., 2008; Xu et al., 2008). The symptoms of 22q11.2-related schizophrenia are indistinguishable from those of idiopathic schizophrenia (Pulver et al., 1994; Murphy et al., 1999; Chow et al., 2006; Antshel et al., 2016; Mollon et al., 2018), suggesting the existence of common mechanisms between the two subtypes of the disease. Recent works have identified more CNVs, such as 1q21.1 (del), 1q21.1 (dup), 2p16.3 (del, containing the *NRXN1* gene), 3q29 (del), 7q11.2 (dup), 15q13.3 (del), and 16p11.2 (dup), that also increase the risk of schizophrenia and can be modeled in mice, rats, flies, and zebrafish (Marshall et al., 2017; Forsingdal et al., 2019).

The high (but not complete) penetrance of CNVs associated with neuropsychiatric disorders (Stefansson et al., 2014) suggests their use in constructing animal models, but with limitations (Forsingdal et al., 2019). Animal models have a uniquely important place in elucidating disease mechanisms, i.e., they make possible the functional investigations of disease in living brains. For animal models to be of translational use, they must satisfy face, predictive, and construct validity criteria (Chadman et al., 2009; Nestler and Hyman, 2010). *Face validity* describes the ability of a model to accurately reproduce important features of the human disease or a specific trait under investigation. The face validity criterion undoubtfully suffers from modeling psychotic or other self-reported symptoms in animals. However, recently progress has been made in measuring hallucination-like percepts in mice (Schmack et al., 2021). *Predictive validity* relates to the ability of successful treatments in people with the disease to ameliorate symptoms in the model. For example, any observable psychosis-related phenotypes in the model should also be reduced by the administration of antipsychotics. However, predictive validity does not account for patients with treatment-resistant disease. In this case, models that cannot be ameliorated by antipsychotics could potentially guide us to identifying novel treatments. *Construct validity* requires that the model be generated by the same etiologic processes (e.g., genes, toxins, infectious agents, stressors) that occur during the disease. Construct validity is often difficult to achieve in preclinical models of schizophrenia because the exact underlying causes of the disease are unknown and do not appear to be homogeneous across the patient population. Recently, the validity requirements for neurodevelopmental disease models were called into question, and it was suggested that face validity and construct validity be abandoned and replaced with a detailed, quantitative phenotypic comparison of human and animal features at relevant levels of analysis (Hyman, 2021).

Copy number variants-based animal models come close to satisfying validity requirements. For instance, 22q11DS mouse models have been constructed by deleting a region of mouse chromosome 16 homologous to the 22q11.2 region in humans (Lindsay et al., 1999; Merscher et al., 2001; Stark et al., 2008; Saito et al., 2020). The murine region contains orthologs of all but one human gene, with only minimal reorganization of gene order (reviewed in Karayiorgou et al., 2010). The 22q11DS mouse models are deficient in sensorimotor gating (Paylor et al., 2001, 2006; Paylor and Lindsay, 2006) and working and emotional memory (Sigurdsson et al., 2010; Piskorowski et al., 2016). Furthermore, mouse models of 22q11DS recapitulate several physiological, morphological, and behavioral phenotypes associated with schizophrenia (Stark et al., 2008; Kvajo et al., 2012; Chun et al., 2014; Piskorowski et al., 2016; Eom et al., 2017; Donegan et al., 2020). Thus, mouse models of 22q11DS are etiologically valid models due to a high degree of conservation with the 22q11.2 region, and these animals recapitulate several anatomical (Lindsay et al., 1999, 2001; Merscher et al., 2001) and behavioral (Paylor et al., 2001; Earls et al., 2010) features of the human disease. Thalamic abnormalities have been also described in 22q11DS mouse models (Chun et al., 2014, 2017; Eom et al., 2017). The auditory thalamocortical abnormalities in these mutant mice (described in the next section of this review) arise later in life and are rescued by antipsychotics (Chun et al., 2014).

Caution is warranted when considering CNV-based models as accurate models of schizophrenia. The effects of CNVs are pleiotropic. For instance, 22q11.2 microdeletion substantially increases the risk of not only schizophrenia but also autism, intellectual disability, Parkinson disease, and others (Stefansson et al., 2014; McDonald-McGinn et al., 2015). Furthermore, they may interact with common risk variants to produce schizophrenia (Bergen et al., 2019; Davies et al., 2020; Cleynen et al., 2021). In addition to these limitations, incomplete synteny in the deletion or duplication of genomic regions, relatively poor conservation of the non-coding regions of the genome across species, and the lack of complete homology between human and mouse brain structures makes it uncertain whether the mutant mice qualify as true translational models of schizophrenia. Therefore, animal models carrying CNVs can be conceptualized as tools with which to study basic mechanisms related to CNV syndromes rather than as models of schizophrenia *per se*.

## Schizophrenia-Risk Genes and Mechanisms of Thalamic Dysfunction

Given the overwhelming evidence of thalamic dysfunction in schizophrenia, it is important to consider how the identified genetic risks could be mechanistically involved in the pathogenic processes. A previous attempt was made to combine identified common risk variants and CNVs into a thalamus-centered model of schizophrenia (Richard et al., 2017). Here, we focus on thalamic aspects of the 22q11.2 microdeletion, a CNV that confers one of the strongest genetic predictors of schizophrenia, and its mechanistic connection to several common gene variants identified in GWAS (Ripke et al., 2014) [e.g., DRD2, SERCA2 (encoded by the *ATP2A* gene) and CAV3.3 (encoded by the *CACNA1I* gene)]. There are indications of abnormal thalamic morphology in individuals with 22q11DS. For instance, those with auditory hallucinations have a smaller volume of the medial geniculate nucleus and hyperconnectivity between the medial geniculate nucleus and the auditory cortex, which was interpreted as a delay or lack of maturation of thalamocortical connectivity (Mancini et al., 2020).

**FIGURE 2:**
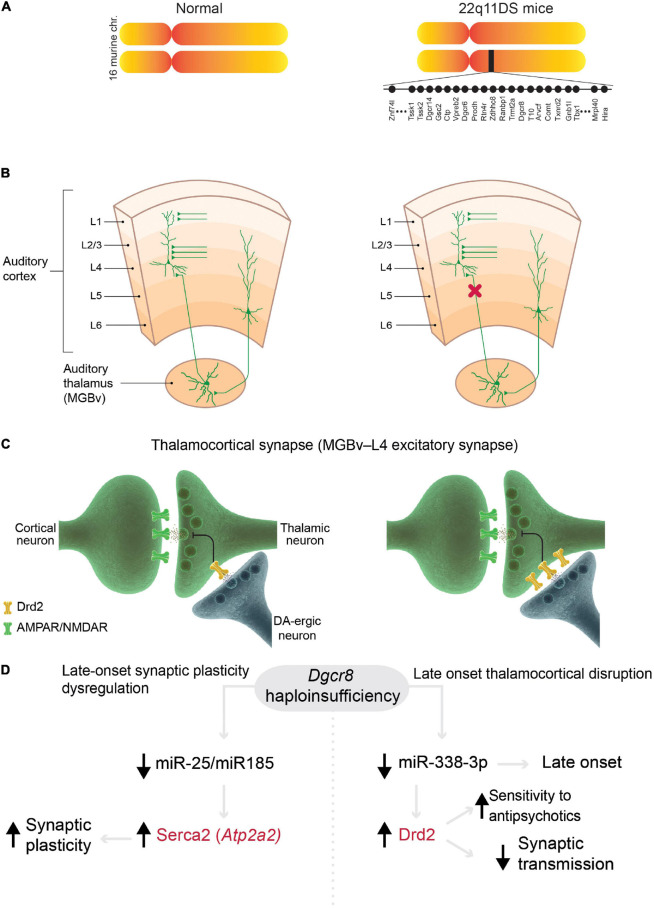
Models of 22q11 deletion syndrome (22q11DS) reveal mechanisms in common with schizophrenia. **(A)** Mouse models of 22q11DS carry a hemizygous microdeletion of the genomic region of mouse chromosome 16 (MMU 16qA13) containing orthologs deleted in the syntenic 22q11.2 locus in humans. Each filled circle represents one gene. **(B,C)** Mechanisms of specific thalamocortical functional disruption in 22q11DS mice. **(B)** Synaptic transmission at excitatory thalamocortical projections between the auditory thalamus [medial geniculate body, ventral division (MGBv)] and the auditory cortex is reduced (decrease in probability of glutamate release from thalamic presynaptic terminals, red X) but other excitatory projections (corticocortical and corticothalamic projections) remain intact in 22q11DS mice. **(C)** The deficit in synaptic transmission in 22q11DS mice is caused by reduced glutamate release from thalamic presynaptic terminals due to upregulation of Drd2 in the thalamic neurons (Chun et al., 2014). DA-ergic neuron, dopaminergic neuron. **(D)** In 22q11DS mice, late-onset deficits in synaptic plasticity (left) and thalamocortical synaptic transmission (right) are caused by *Dgcr8* haploinsufficiency and miRNA depletion. Abnormal synaptic plasticity depends on age-dependent dysregulation of Serca2 in 22q11DS, which was originally described in hippocampal synapses (Earls et al., 2010, 2012). This mechanism presumably affects most excitatory synapses in the brain. This age-dependent mechanism was described in auditory thalamocortical synapses (Chun et al., 2017). Mutations in *ATP2A2* (encoding SERCA2) and *DRD2* (in red) were identified as common risk variants in schizophrenia (Ripke et al., 2014).

The mouse models of 22q11DS that contain the microdeletions of the syntenic region (Figure 2A), which is associated with psychosis in humans (Karayiorgou et al., 2010; McDonald-McGinn et al., 2015), have several abnormalities related to the positive symptoms. First, thalamocortical projections to the auditory cortex are disrupted in 22q11DS mice (Chun et al., 2014). This disruption is functional rather than anatomical, i.e., activity-dependent glutamate release from presynaptic (thalamic) terminals is decreased (Figures 2B,C). This functional disruption is specific to thalamocortical projections in the auditory cortex; other thalamocortical projections, such as to the visual and somatosensory cortices and the corticocortical, corticothalamic, or hippocampal projections, remain intact. Second, the auditory thalamocortical disruption in 22q11DS mice is rescued by antipsychotics (haloperidol, clozapine, olanzapine) and specific DRD2 inhibitors (Chun et al., 2014, 2017). This is explained by an aberrant elevation of Drd2s in the thalamus, which renders 22q11DS thalamocortical projections hypersensitive to antipsychotics (Figures 2C,D). It has been suggested that aberrant elevation of Drd2s may hyperpolarize thalamic neurons by activating potassium channels (Lavin and Grace, 1998), thus promoting delta oscillations (Richard et al., 2017). Third, the onset of thalamocortical disruption in 22q11DS mice appears at around 3.5–4 months of age (Chun et al., 2017), a developmental stage in mice that corresponds to young adulthood in humans (Flurkey et al., 2007). This mechanism integrates several competing schizophrenia theories, such as dopamine hyperactivity or hyperfunction hypothesis (Carlsson, 1988) (because the Drd2 expression level is abnormally elevated in the thalamus of 22q11DS mice), glutamatergic hypofunction hypothesis (Coyle, 1996, 2006) (because disruption occurs at glutamatergic thalamocortical synapses), thalamocortical dysconnectivity hypothesis (Friston and Frith, 1995; Woodward et al., 2012), and thalamocortical loop dysfunction hypothesis (Behrendt, 2003).

The thalamocortical abnormalities in 22q11DS mice are caused by the haploinsufficiency of the *Dgcr8* gene (Chun et al., 2014; Figure 2D), which is encoded within the microdeletion and is a part of the microprocessor complex that mediates the biogenesis of microRNAs (miRNAs), small non-coding RNAs that bind target mRNAs by complementary base pairing and inhibit mRNA translation or promote mRNA degradation (Ambros, 2004). *Dgcr8* haploinsufficiency in 22q11DS leads to depletion of thousands of miRNAs and the resultant upregulation of multiple targets, which in turn, disrupts or otherwise alters synaptic transmission, synaptic plasticity, and proper functioning of multiple neural circuits responsible for different behaviors (Earls and Zakharenko, 2013). Thus, the haploinsufficiency of one 22q11DS gene, *Dgcr8*, may result in pleiotropic effects on various neural circuits through depletion of different sets of miRNAs, whose expression varies across cell types and development (Earls et al., 2014). For instance, thalamocortical disruption in 22q11DS mice was mediated by *Drd2*-targeting miR-338-3p (Figure 2D), whose expression profile may explain the late onset and specificity of auditory thalamocortical disruption (Chun et al., 2017). In another instance, *Dgcr8* haploinsufficiency in 22q11DS mice led to the depletion of miR-25 and miR-185, the upregulation of Serca2 protein (encoded by *Atp2a2*), and an age-dependent increase in synaptic plasticity (Earls et al., 2010, 2012; Figure 2D). Serca2 is a calcium pump that is expressed throughout the brain, including the thalamus, hippocampus, and cortex (Lein et al., 2007). Serca2 serves an important role in delta-oscillation generation (Cueni et al., 2008), and *ATP2A2* mutations are associated with psychosis (Nakamura et al., 2016; Takeichi et al., 2016; Gordon-Smith et al., 2018). Most notably, mutations in both *DRD2* and *ATP2A2* have been identified by GWAS as common risk variants in schizophrenia (Ripke et al., 2014). This suggests that these molecules (and possibly many others regulated by the *Dgcr8*–miRNA-dependent mechanism) serve as a common mechanistic node between schizophrenia cases arising from the 22q11.2 microdeletion and idiopathic cases of the disease (without CNVs).

The 22q11.2 microdeletion, as a risk factor of schizophrenia, may act through haploinsufficiency of *DGCR8* and depletion of downstream miRNAs. Idiopathic schizophrenia is also strongly linked to miRNAs. Most notably, genetic variants of *MIR137*, which encode miR-137, contribute to schizophrenia risk (Ripke et al., 2011, 2013, 2014; Thomas and Zakharenko, 2021). Other miRNAs identified by GWAS have been implicated in schizophrenia (Yoshino and Dwivedi, 2020). Schizophrenia-associated miRNAs may act synergistically to mediate disease risk. It has been suggested that the loss of function in multiple miRNAs may lead to psychiatric disease (Thomas et al., 2018). It is conceivable that another common mechanistic node between idiopathic schizophrenia and those cases arising from the 22q11.2 microdeletion may be a depletion (or loss of function) of subsets of miRNAs in the thalamus.

Another notable common risk variant identified in schizophrenia and related to thalamic function is *CACNA1I* (or CaV3.3), which encodes a T-type calcium (Ca^2+^) channel (Ripke et al., 2014; Narayanan et al., 2015). *CACNA1I* is predominantly expressed in the inhibitory neurons of the TRN. As mentioned above, thalamic excitatory neurons and TRN inhibitory neurons are reciprocally connected, and this complex network contributes to thalamocortical oscillations (Crunelli et al., 2014). Oscillatory activity originating in the thalamus is regulated by T-type Ca^2+^ channels (Beurrier et al., 1999; Cain and Snutch, 2013). CaV3.3 is thought to be necessary for generating the low-threshold calcium spikes required for generation of low-frequency oscillations in the TRN neurons (Astori et al., 2011; David et al., 2013). In individuals with schizophrenia, *de novo* missense variations in genes encoding T-type Ca^2+^ channels can impede the function of the channels by reducing Ca^2+^ currents and decreasing the bursting ability of thalamic projection neurons (Gulsuner et al., 2013; Andrade et al., 2016). Moreover, antipsychotics can target voltage-gated Ca^2+^ channels, and antagonists of T-type Ca^2+^ channels demonstrate antipsychotic effects (Kraus et al., 2007; Kopecky et al., 2014). For instance, clozapine, a second-generation antipsychotic, binds T-type Ca^2+^ channels and restores the ability of the cell to fire in bursts (Choi and Rhim, 2010).

Slow (delta/theta) thalamocortical oscillations are typically associated with non-rapid eye movement (NREM) sleep states. Sleep spindles, a hallmark of stage 2 NREM sleep, are thalamic in origin, with TRN neurons powerfully modulating thalamocortical activity through rhythmic inhibition (Steriade et al., 1985, 1993; Pinault, 2004). Clinical studies have shown that individuals with schizophrenia have difficulties initiating sleep (Poulin et al., 2003), and encephalographic recordings of their sleep show fewer sleep spindles (Ferrarelli et al., 2007; Young and Wimmer, 2017; Manoach and Stickgold, 2019). These findings support the hypothesis that disruptions of sleep in people who have schizophrenia are caused by deficient TRN inhibition and, in turn, disrupted thalamocortical oscillations. Whether CaV3.3 is associated with these endophenotypes is unclear. However, several synergistic mechanisms that converge on the thalamic circuitry may underlie abnormal oscillatory activity in schizophrenia. For instance, mechanisms that promote hyperpolarization of thalamic relay neurons and cause T-type Ca^2+^ channel spikes (i.e., the *Dgcr8–miR-338-3p*–Drd2 mechanism in 22q11DS or CaV3.3-dependent TRN inhibition) may synergistically produce sufficient hyperpolarization to activate T-type Ca^2+^ channels, which in turn would cause the channels to produce abnormal delta-frequency oscillations (Richard et al., 2017).

The thalamus is also essential for cognition (reviewed in Rikhye et al., 2018b). Work in mice has shown that thalamocortical projections are critical for encoding cognitive functions that are affected in schizophrenia. For instance, reciprocal activity between the mediodorsal nucleus of the thalamus and the prefrontal cortex is required for maintenance of spatial working memory, cognitive flexibility, adaptive decision making, and sustaining attentional control in rodents (Bolkan et al., 2017; Schmitt et al., 2017; Alcaraz et al., 2018; Rikhye et al., 2018a; Mukherjee et al., 2021). However, the role of these thalamocortical circuits in cognitive symptoms has yet to be established in mouse models of 22q11DS or other schizophrenia-associated CNVs. The working memory dysfunction in 22q11DS mice has been attributed to hippocampal dysfunction or deficient oscillatory activity between the hippocampus and the prefrontal cortex (Sigurdsson et al., 2010; Devaraju et al., 2017). Together, results from human and mouse model studies reinforced each other and led to the emergence of new hypotheses that can be tested in animal models. Below we describe several such hypotheses and their relevance to the thalamocortical circuits.

## Overarching Hypotheses of Positive Symptoms in Schizophrenia: Thalamic Involvement

The most prominent theories of schizophrenia symptoms include the dopamine hyperfunction hypothesis (Carlsson, 1988), the glutamate hypofunction theory (Coyle, 1996, 2006), and the disconnectivity hypothesis (Friston and Frith, 1995; Woodward et al., 2012). As mentioned above, these theories mechanistically converge in the thalamus as late-onset synaptic disruption of thalamocortical projections is observed in 22q11DS mice (Chun et al., 2014, 2017). More recently, other theories of psychosis have emerged, some linked to the striatum and basal ganglia (Horga and Abi-Dargham, 2019) as the brain regions most heavily regulated by dopamine and with the highest expression levels of dopamine receptors, including DRD2s (De Keyser et al., 1985; Palacios et al., 1988). These theories are based on integration of neural circuits from multiple brain regions and may involve thalamic participation [perhaps via cortico–striatal–thalamocortical loops (Alexander et al., 1986; Haber, 2003) or via a thalamo–hippocampal–ventral tegmental area loop (Lisman et al., 2010)].

Without accurate and finely controlled thalamic processing, our ability to distinguish between real and false perceptions may fail, and this failure may be the root of hallucinations. As explained by Bayesian brain theory, the brain is a statistical organ that generates hypotheses about the environment based on prior expectations. Previously generated expectations are incorporated into the decision-making process, such that prior knowledge provides an estimate for the prior beliefs held about an external signal, which then dictates the probability of certain behavioral responses (reviewed in Horga and Abi-Dargham, 2019). This model relies on predictive coding: a neural representation at the cortical level that predicts representations at lower levels. These top–down predictions are compared with bottom–up representations to form a prediction error or mismatch signal. The mismatch signal is then sent back up the hierarchy to update the sensory representations (Friston et al., 2016). To develop an accurate representation, the brain needs to correctly infer the content of the sensory inputs. It is hypothesized that these ascending projections essentially deliver “newsworthy” information that, in turn, is explained by descending predictions.

The Bayesian brain hypothesis can be likened to a neurobiological phenomenon known as “corollary discharge” (Crapse and Sommer, 2008; Sommer and Wurtz, 2008). Animals need to be able to distinguish between self-generated sensory inputs and those caused by the external environment. In corollary discharge, a copy of a motor command is projected to sensory areas of the brain to inform the system of a self-generated movement and, therefore, a self-generated sensory experience. This recursive signal is critical to inhibit reflexes generated by an animal’s own movements and plays a key role in updating external representations within the brain. The idea that the inability to distinguish one’s self from environmental inputs (i.e., a lack of corollary discharge) underlies psychotic symptoms has been proposed (Heinks-Maldonado et al., 2007; Ford et al., 2008; Parlikar et al., 2019). Under this theory, thalamocortical projections send parallel streams of information to cortical and subcortical regions, most likely creating a corollary discharge–like predictive model of sensory experiences. Therefore, the corollary discharge signal may function as one anatomical correlate to the Bayesian brain hypothesis. The brain is constantly monitoring sensory perceptions and motor responses, essentially creating predictive models of the external world. This model also extends to the predicted responses that occur when an animal interacts with its environment. If a mismatch or prediction error occurs within this system, possibly due to faulty thalamic signaling, a false perception, such as a hallucination or delusion, will most likely occur.

It has also been suggested that schizophrenia is a disorder of consciousness (Frith, 1979; Anscombe, 1987; Giersch and Mishara, 2017). The dendritic integration theory proposed by Larkum and colleagues is a cell-based theory about how the brain encodes perception and consciousness. This theory may help link multiregional hypotheses by providing a specific cellular mechanism (Aru et al., 2019, 2020; Bachmann et al., 2020). The dendritic integration theory is based on the *trans*-thalamic model proposed by Sherman and Guillery (2013) and posits that bottom–up signals arising from first-order and higher-order thalamic inputs converge with top–down cortical inputs onto the dendritic arbors in layer 5 pyramidal cells. These projections terminate in distinct dendritic regions, with bottom–up inputs synapsing onto basal dendrites and top–down inputs targeting apical dendrites. Temporally coincident activity from these inputs induces burst firing in layer 5 pyramidal cells through active dendritic integration, acting as a cellular mechanism for associative pairing by linking external stimuli with internal representations. This dendritic integration occurs across the cortical mantle, encompassing multiple sensory modalities and executive functions. The dendritic integration theory also provides a potential cellular mechanism to explain how the thalamocortical system functions as a predictive organ. If incoming signals from top–down and bottom–up sources are not temporally coordinated onto layer 5 dendritic arbors, then a mismatch or prediction error signal may develop. Together, Bayesian brain theory and dendritic integration theory predict vulnerabilities of the thalamocortical system that may play an important role in schizophrenia. These predictions are now poised for testing in etiologically relevant animal models of schizophrenia.

## Conclusion

The unique architecture of first-order and higher-order thalamic nuclei generates a vast interconnected network that propagates selective incoming sensory information to multiple cortical regions. First-order nuclei send thalamocortical projections to specific, confined cortical zones, whereas higher-order thalamic nuclei radiate processed information by broadly innervating several cortical regions. Thus, information arising from one modality is integrated across cortical and thalamic regions through a vast oscillating network of recurrent thalamocortical activity. Because of the rich interconnectedness of thalamic nuclei with various cortical (e.g., sensory, prefrontal cortices) and subcortical (e.g., hippocampus, striatum, amygdala) regions, the thalamus is uniquely positioned to explain processing abnormalities, such as hallucinations, as well as cognitive and negative symptoms of schizophrenia.

Oscillatory activity occurs within the thalamocortical loops, and that information from a single modality is broadcast across multiple cortical and subcortical regions via *trans*-thalamic circuits (Shepherd and Yamawaki, 2021). Moreover, these pathways send duplicate copies of motor commands through parallel projections, most likely creating a prediction signal. Disruptions within the prediction signal may generate false perceptual experiences or disturbances in cognitive processing, two of the hallmark symptoms in schizophrenia. Thus, seemingly disparate schizophrenia symptoms may be caused by a common mechanism: disruption within the thalamus-processing network. Given our recently gained knowledge of the genetic risk variants for schizophrenia, these predictions can be mechanistically tested in models carrying mutations carefully designed to match the human condition. This direction shows great potential given the progress in behavioral phenotyping translationally relevant psychotic, motivational, and other symptoms in rodents (Simpson et al., 2012; Schmack et al., 2021). Furthermore, mechanistic studies of thalamic dysfunction may soon be spurred by remarkable progress in the development of the thalamic neuronal 3-dimensional cultures [i.e., organoids (Xiang et al., 2019) and thalamocortical assembloids (Pasca, 2018; Xiang et al., 2019)] derived from reprogrammed human cells (Khan et al., 2020). For example, neuronal or synaptic abnormalities in thalamocortical or corticothalamic projections in assembloids derived from individuals with schizophrenia or 22q11DS could potentially inform us about new human-specific therapeutic strategies. Although the latter approach alone is problematic, as it cannot model the late onset of schizophrenia (organoids mostly consist of immature neurons), a combinatorial approach using both animal models and human organoid models within the established theoretical frameworks promises a way forward in our mechanistic understanding of schizophrenia.

## Author Contributions

YJ and MHP wrote the first draft of this review manuscript. SSZ wrote the final version. All the authors contributed to the article and approved the submitted version.

## Conflict of Interest

The authors declare that the research was conducted in the absence of any commercial or financial relationships that could be construed as a potential conflict of interest.

## Publisher’s Note

All claims expressed in this article are solely those of the authors and do not necessarily represent those of their affiliated organizations, or those of the publisher, the editors and the reviewers. Any product that may be evaluated in this article, or claim that may be made by its manufacturer, is not guaranteed or endorsed by the publisher.
